# The Design of High Performance, Low Power Triple-Track Magnetic Sensor Chip

**DOI:** 10.3390/s130708771

**Published:** 2013-07-09

**Authors:** Xiulong Wu, Minghua Li, Zhiting Lin, Mengyuan Xi, Junning Chen

**Affiliations:** 1 School of Electronic and Information Engineering, Anhui University, Hefei 230601, China; E-Mails: xiulong@ahu.edu.cn (X.W.); xmy_1314@126.com (M.X.); jnchen@ahu.edu.cn (J.C.); 2 Department of Electrical Engineering, University of Texas at Dallas, Richardson, TX 75080, USA; E-Mail: mxl095420@utdallas.edu

**Keywords:** triple-track, magnetic sensor, decoder

## Abstract

This paper presents a design of a high performance and low power consumption triple-track magnetic sensor chip which was fabricated in TSMC 0.35 μm CMOS process. This chip is able to simultaneously sense, decode and read out the information stored in triple-track magnetic cards. A reference voltage generating circuit, a low-cost filter circuit, a power-on reset circuit, an RC oscillator, and a pre-decoding circuit are utilized as the basic modules. The triple-track magnetic sensor chip has four states, *i.e.*, reset, sleep, swiping card and data read-out. In sleep state, the internal RC oscillator is closed, which means that the digital part does not operate to optimize energy consumption. In order to improve decoding accuracy and expand the sensing range of the signal, two kinds of circuit are put forward, naming offset correction circuit, and tracking circuit. With these two circuits, the sensing function of this chip can be more efficiently and accurately. We simulated these circuit modules with TSMC technology library. The results showed that these modules worked well within wide range input signal. Based on these results, the layout and tape-out were carried out. The measurement results showed that the chip do function well within a wide swipe speed range, which achieved the design target.

## 1. Introduction

### 1.1. Conception of Magnetic Card Reader

Magnetic cards are widely used in modern society. They are small, thin, and light, which makes it possible for us to carry many of them in our wallets. In recent years, magnetic cards are used for cash cards, patient registration cards, membership cards, security cards and the like. A long and narrow magnetic stripe embedded in a surface of a magnetic card has digital data such as a number for identifying a user, magnetically recorded therein [1-3]. Depending on the different materials, magnetic cards can be divided into three kinds: PET cards, polyvinyl chloride cards and paper card. The majority of magnetic cards are constructed using a substrate of polyvinyl chloride. The magnetic stripe is laminated onto the card such that its surface is flush with the rest of the PVC card. Complying with ISO 7811.2 standards, the magnetic stripe is 10.3 mm wide in order to allow the presence of three tracks (ISO 7811.4 & 7811.5) and is located 5.0 mm from the top reference edge of the card. The magnetic stripe length is generally the length of the card (defined by ISO 7810 standard) [4]. And from another point of view, the magnetic cards can be also divided into the magnetic stripe cards and full-coated magnetic cards depending on different magnetospheric structures. Now, current magnetic cards have a magnetic strip that is divided into three tracks. Track 1 uses a format established by the Air Transport Association and normally stores a name. Track 2 was defined by the banking industry and typically stores an account number (e.g., a credit card number). Track 3 was originally intended for use with Automatic Teller Machines [5].

The magnetic card decode chip is a sensor actually. This chip acts as a kind of bridge between the magnetic head and the micro-processor. It can change the signals sensed from a magnetic head into output voltages to meet the need for transmission, and storage. The distance between the magnetic gap and the magnetic recording medium is called the spacing. When the spacing is large both recording and reproducing efficiencies fairly deteriorate [6]. To write data to a magnetic card, we need a magnetic write head and an electronic current drive circuit capable of magnetizing the magnetic oxide in the magnetic stripe to full magnetization (saturation). Generally, the recording magnetic head is composed of an annular iron core with a gap therein and a coil wound around the iron core. The encoding current in the write head is capable of alternating direction, producing alternating zones of magnetization direction on the magnetic stripe. Therefore, the data information can be encoded on the magnetic stripe [7].

**Figure 1.**
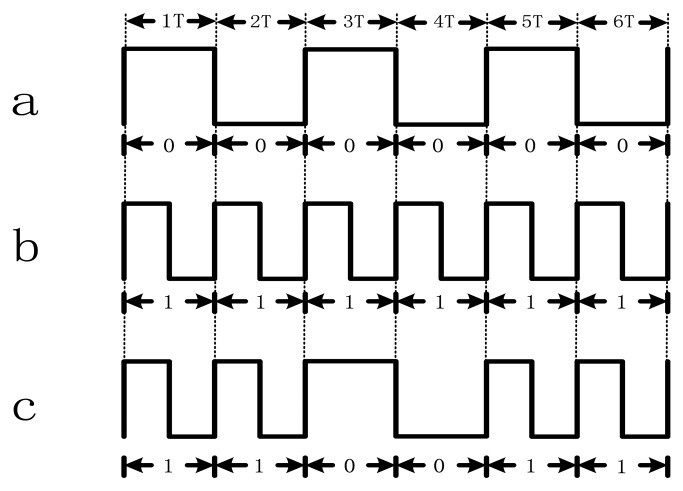
F2F coding rule. (**a**) If there is no flux in one period (T), then the signal is recorded as “0”. (**b**) If there is a flux in one period (T), then the signal is recorded as “1”. (**c**) The signal presents 6-digit binary number “110011”. In other words, F2F coding depends on the flux of current instead of the absolute current value. As shown in part a, although the signal is positive in T1, and negative in T2, we recorded them as two “0”s, because there's no toggle within each T.

The efficient methods of encoding includes: frequency modulation (FM), phase modulation system (PM), and double frequency phase coherence recording (F2F). In FM recording mode, writing information is implemented by changing the frequency of write current. F2F coding is an improved single coding method based on the FM frequency modulation. Flux transition generated between two periods is denoted by “1” (as shown in Figure 1a), and no flux transition is denoted by “0” (as shown in Figure 1b). Generally, unscrambling data is accomplished by distinguishing relative pulse widths of the data bits. To present 6-digit binary number “110011”, the write current shown in Figure 1c is implemented.

The data reading is a reverse process of data writing. The major function of triple-track magnetic sensor chip is to sense the data stored in magnetic cards. The magnetic information of a card is read while the card is moved along a groove-shaped card passage, where contains the magnetic heads [8]. The read head converts the changing of magnetic flux in the coil of the read head into a voltage pattern mirroring the magnetization zones of the encoded data. The voltage pattern then can be translated by the decoding electronics into the binary data [7].

### 1.2. Design Target

Before the chip design, we analyzed some similar design. BS730 is a CMOS ASIC IC for F2F magnetic stripe card reader. It is a 24 pin triple-track IC, which can operate at the range from -10 to 55 temperature (°C) with only 4 external capacitors [9]. AIS-2103N is used to recover F2F encoded data received from a magnetic head. It is commonly used in Point-of-Sales terminal and electronic money transfer terminal. It is a 32 pin triple-track IC, which requires 6 external resistors and 8 external capacitors [10]. SMR200 provides decoding function for magnetic stripe storage system, with all the analog and digital circuits in a single chip. F2F pattern signal is generated by analog signal processing through an amplifier OP1, peak detector OP2 and comparator. The operation of digital logic for data generation is activated by triggering oscillation circuit as soon as detecting F2F pattern transition. The standby current is only 0.8 mA. It is a 24 pin dual-track IC, which requires 6 external resistors and 8 external capacitors [11]. M3-2200G can recover clock and data signals from an F2F data stream generated from a magnetic head. M3-2200G is consisted by two major blocks at each channel, *i.e.*, amplify block and control block. Amplify block amplifies and filters the signal read from the magnetic reader head, rejects common mode noise and detects signal peaks. The enable and disable counters in control block provide initialization for recovery block. These counters initialize both bit recovery and the signal conditioning and detection block. It is a 28 pin dual-track IC, which requires 13 external resistors and 11 external capacitors [12].

Based on the analysis of the performance of the existing chips, we proposed an 8 pin triple-track IC, which requires no external resistors and capacitors. The chip can work properly at the range from -30 to 80 temperature (°C). When the voltage is between 2.7 V to 3.6 V, the magnetic card can read and write data at the swiping speed within a wide swipe speed range. Magnetic cards may be old or new, and the magnetic attenuation may occur due to improper storage or using. So the chip has to be made to read the data from 30% to 200% of International Standards Organization (ISO) 7811 amplitude standard. The differences of these 5 chips are summarized in Table 1.

The contribution of this work is to provide a new solution for implementing a small outline, high performance, and low power triple-track magnetic sensor chip. The pin number is eight, which is much smaller than other four chips. It requires no external components, and can be built inside magnetic heads. Besides, it consumes less than 120 μA, when the sensor chip is held in the sleep state. It is suitable for portable, hand-held devices.

There are two ways to enhance the performance of the triple-track magnetic sensor chip. One of which is to improve the performance of digital processing algorithm. It can reduce the accuracy requirements of the decoding clock, and improve the capability of error correction. Another method is to improve the performance of the signal sensing by correcting the offset of the signal amplifier and improving the accuracy of pre-decoding circuits. In this paper, we mainly focus on the second method. We put forward two circuits, *i.e.*, the offset correction circuit and the tracking circuit. These circuits can correct the offset of the signal amplifier, enlarge the sensing range of the magnetic stripe signal, and improve the pre-decoding capability.

## 2. Overview of the Triple-Track Magnetic Sensor Chip

### 2.1. The Card Swiping System

**Figure 2.**
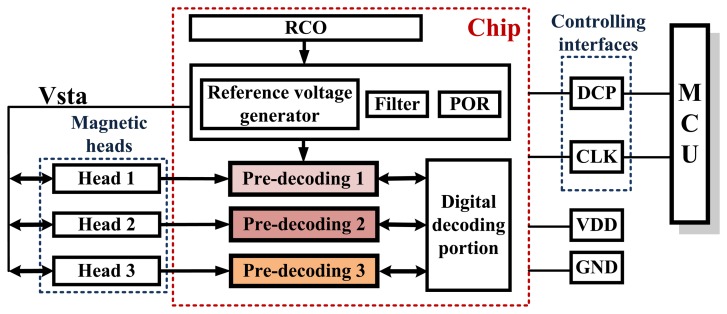
Diagram of the system architecture. The card swiping system is composed of read heads, a triple-track magnetic sensor chip and the controlling interfaces. The triple-track magnetic sensor chip contains RCO, RC filter, POR, the reference voltage generating circuit, pre-decoding circuits and digital decoding circuits.

The swiping system is composed of read heads, a triple-track magnetic sensor chip, power supply (VDD), GND and two controlling interfaces (DCP and CLK). The sensor chip is made up of some basic circuit modules, such as: voltage reference, filter, power-on reset (POR), pre-decoding circuit, an RC oscillator (RCO) and the digital decoding circuits. The controlling interfaces include two ports. One is the data_control inputoutput port (DCP), which is used for exporting the sensed data and inputting the control signal; and another is the clock input interface (CLK), which is a synchronization signal for extracting data from the ASIC. Through these two controlling interfaces, the triple-track magnetic sensor chip can export data to a micro-processor under its control (as shown in Figure 2).

Because the magnetic cards are usually swiped manually, the swiping speed is unstable, which will lead to static interference, followed by the increasing of the signal glitch. Hence the internal RC filter is used to shape the signal waveform. POR module is introduced to provide a power-on reset signal to reset the digital circuits. The bandgap voltage reference can generate the desired reference voltages and currents with very small temperature coefficient, and create a standard voltage *V_sta_* for comparison with the sensed signals.

### 2.2. The Structure of the Pre-Decoding Circuit

**Figure 3.**
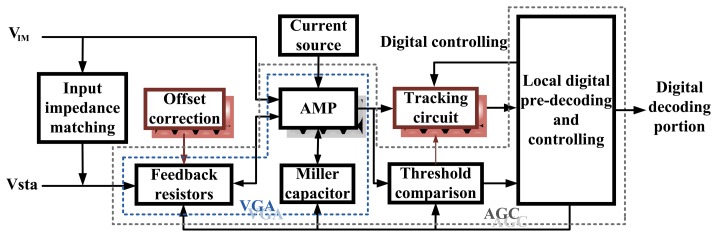
Pre-decoding circuit architecture diagram. The amplified sensed signal flows into the tracking circuit and the threshold compare circuit module, and is decoded by a local digital pre-decoding circuit.

Three tracks of the pre-decoding circuit are all the same in terms of the structure. Each pre-decoding circuit module is composed of an impedance matching circuit, feedback resistors, Miller capacitances, a low power amplifier, an offset correction circuit, a simple local digital pre-decoding and controlling circuit. The basic circuit structure of the pre-decoding circuit is shown in Figure 3.

Automatic Gain Control (AGC) loops are used to maximize the dynamic range of overall systems in medical equipments, telecommunication systems, hearing aids, disk drives, and others [13–15]. The main goal of the AGC loops is to automatically control its gain in response to the amplitude of the input signal, leading to a constant-amplitude output. So that downstream circuits require less dynamic range [16]. One of the key circuit elements involved in an AGC loop system is the variable gain amplifier (VGA) [17]. Several feedback resistors are used to adjust the gain of the amplifier, and the Miller capacitances are used to compensate the amplifier. The sensed signal *V_IM_* along with *V_sta_* through feedback resistors are connected to the differential inputs of the amplifier. The output of the amplifier is the input of the tracking circuit. The sine wave is converted into pulse wave to reflect the mutation of the input waveform. Only if the slope of the input waveform has a mutation, will there produce a changed output signal, otherwise, the output is constant. The output voltage of the amplifier is also the input of the threshold comparator circuit. The comparator circuit generates a feedback control signal to adjust the value of Miller capacitor and the ratio of the feedback resistors, which can adjust the bandwidth and the gain of the amplifier. Tracking circuit and the threshold comparison circuit generate some signals for controlling the digital pre-decoding circuit, meanwhile the local digital controlling circuit also generates a set of digital signals to turn on/off some of the analog circuits. Note that the inherent offset may affect the performance of the AGC, and F2F decoding depends on the fluxes of voltages instead of the absolute voltage value. Therefore, we propose two types of circuits: offset calibration circuit and input tracing circuit, which detailed description will be discussed in Sections 3 and 4.

### 2.3. Operation of the Sensor Chip

The triple-track magnetic sensor chip has four states, *i.e.*, reset, sleep, swiping card and data read-out.

(1) **Reset state.** Once the power supply is turned on or the data readout is over, the system will automatically self-refresh and launch the impedance matching operation.
(2) **Sleep state.** After the impedance matching operation, the sensor chip enters into sleep state and waits for swiping. The internal RC oscillator is closed to optimize energy consumption, which means that the digital decoding portion does not operate. But part of the analog circuits is open to sensor external signal.
(3) **Swiping card.** Once swiping the magnetic card, the internal RC oscillator is turned on and the digital decoding portion begins to work. According to the analog input signals, digital parts automatically adjust the parameter of analog circuits, such as the comparison voltage and the gain of amplification circuit. The signal is amplified to a proper level by adjusting the gain of the VGA. When the swipe speed is too high, or the magnetism of the magnetic card is too strong, the gain of the VGA is automatically reduced to avoid full scale output. When the swipe speed is too low, or the magnetism of the magnetic card is too weak, the sensed signal will be close to the noise, which will eventually affect the decoding. The gain of the VGA is automatically increased. The ideal output signal level should be approximately 1.6 Vpp. The output is connected to the input of the threshold voltage comparator, and is compared with three different threshold voltages. The comparison results is utilized as feedback to control the Miller capacitors and feedback resistors, *i.e.*, to adjust the gain and bandwidth of the amplifier. Meanwhile the output of the VGA flows into the tracking circuit, in which the sine wave is converted to a pulse wave. After sensing all information stored in the magnetic recording medium correctly, DCP interface issues a read end signal. The data will be stored in the internal low cost, low power RAM, at the same time.
(4) **Data read-out.** As mentioned before, when the swiping is over, the DCP issues a read end signal to notify an external MCU. When detecting the read end signal, the MCU can read out the data stored in the internal RAM from DCP by inputting synchronous signal through the CLK interface. After data read out, system reenters into reset state.

## 3. Offset Correction Circuits

The voltage *V_sta_* generated by the reference voltage generating circuit is fed into the triple-track pre-decoding circuits through magnetic heads. Ideally, the differential input of the amplifier is zero. However, offset voltage is inevitable, which causes the output to be much larger or smaller than the required output *V_sta_*. So it's necessary to correct the offset to make the output voltage within a tolerance range of plus or minus 10 percent.

**Figure 4.**
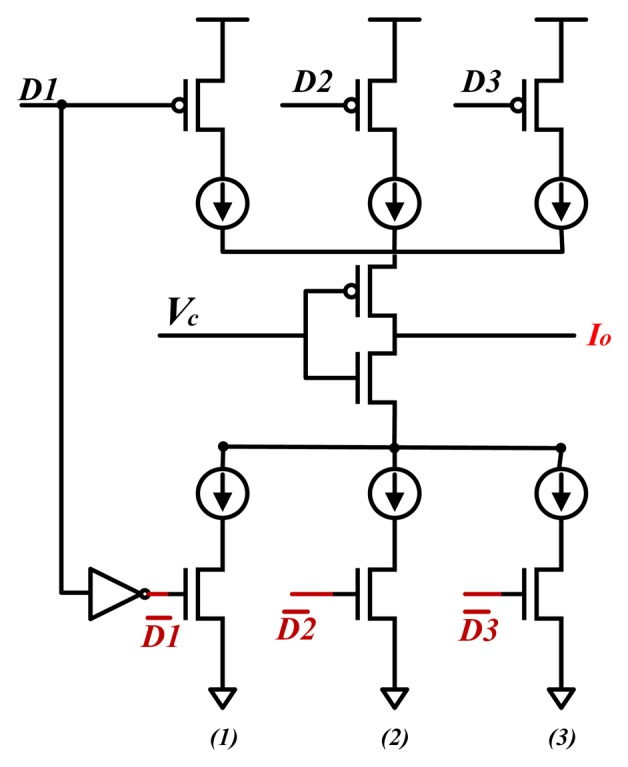
The structure of the current source for offset correction. *D*1, *D*2, *D*3, $\bar{D1}$, $\bar{D2}$ and $\bar{D3}$ are used to control three pairs of PMOS & NMOS transistors. When *D*1 is low, the PMOS transistor is on. $\bar{D1}$ is generated from *D*1, so $\bar{D1}$ is high. The corresponding NMOS transistor is on simultaneously. If *D*1 is high, and $\bar{D1}$ is low, the left group of the PMOS and NMOS is off. When *V_c_* and *D*1 are low, the PMOS of the left group is on, *i.e.*, current generated from upper current sources flows to line *I_o_*. When *V_c_* and $\bar{D1}$ are high, the NMOS of the left group is conductive, *i.e.*, current generated from low current sources flows to line *I_o_*.

Therefore, we propose a current source for offset correction, which contains three pairs of PMOS&NMOS transistors (as shown in Figure 4). The gate voltages of the PMOS and NMOS transistors are *D*1, *D*2, *D*3, $\bar{D1}$, $\bar{D2}$ and $\bar{D3}$. The signals $\bar{D1}$, $\bar{D2}$ and $\bar{D3}$ are generated from the signals *D*1, *D*2, *D*3 by three inverters, respectively. These pairs of digital signals can control the series current sources on or off by making pairs of PMOS&NMOS transistors ON or OFF. Groups of current sources are arranged in side-by-side parallel relationship. So zero or more current sources can be connected to the line *I_o_* in parallel. The selection of the upper current sources or the lower ones is decided by the value of *V_c_*. If *V_c_* is low and at least one of the PMOSs is on, current generated from upper current sources flows to line *I_o_*. If *V_c_* is high and at least one of the NMOSs is on, current generated from lower current sources flows to line *I_o_*.

**Figure 5.**
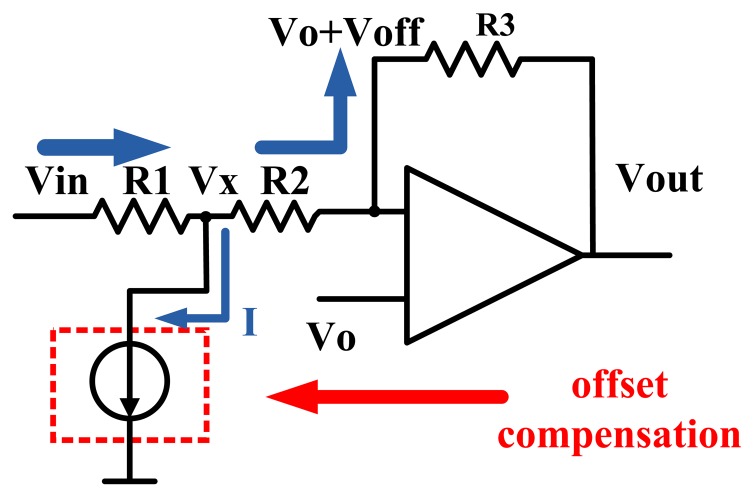
Offset correction circuit diagram. The additional current source provides a small quantity of current *I* to correct the offset, so that the amplifier's output can be much closer to the ideal value.

The whole offset correction circuit diagram is as shown in Figure 5. The additional current source in the red dashed box represents the current source for offset correction as depicted in Figure 4. In other words, we can adjust the amount of the current *I* by controlling the voltage *V_c_* and gate voltages of the PMOS&NMOS transistors as shown in Figure 4. The direction of current *I* is assumed as shown in the Figure 5. One can easily obtain the following two equations according to the Kirchhoff's current law and the Ohm theorem.

(1) $$\frac{V_{in}-V_{x}}{R_{1}}=I+\frac{V_{x}-V_{o}-V_{\text{off}}}{R_{2}}$$

(2) $$V_{o}+V_{\text{off}}-V_{\text{out}}=R_{3}\frac{V_{x}-V_{o}-V_{\text{off}}}{R_{2}}$$

Equation (3) can be derived from Equations (1) and (2):

(3) $$I=\frac{V_{in}}{R_{1}}+\frac{V_{\text{out}}}{R_{2}+R_{3}}-\left(\frac{1}{R_{1}}+\frac{1}{R_{2}+R_{3}}\right)V_{x}$$

and we rewrite Equation (2) in the following form:

(4) $$V_{x}=V_{o}+V_{\text{off}}+(V_{o}+V_{\text{off}}-V_{\text{out}})\frac{R_{2}}{R_{3}}$$

This circuit intends to reach the ideal situation *V_in_* = *V_o_* = *V_out_*, so put this condition into Equations (3) and (4):

(5) $$I=-\left(\frac{R_{1}+R_{2}+R_{3}}{R_{1}R_{3}}\right)V_{\text{off}}$$

From the above formulas we can find that *I* is only related to the offset voltage and the resistors. We performed simulation experiments to analyze the performance of offset correction circuit. The results are summarized in Table 2.

The values of *V_in_* were set to 1.15 V, 1.27 V and 1.39 V, and the ideal output of the VGA were 1.15V, 1.27 V and 1.39 V, respectively. However, because the inherent input offset voltage *V_off_* of the circuit was 0.004 V, the outputs without the offset correction circuit, *V_out_*_1_, were 1.329 V, 1.449 V and 1.569 V, which were much higher than the ideal output voltages 1.15 V, 1.27 V and 1.39 V. And the errors Δ*V_out_*_1_ were 15.5%, 14.1% and 12.9%. If the offset correction current source was introduced in the VGA, the output voltages *V_out_*_2_ came out as 1.08 V, 1.216 V and 1.352 V, which were closer to the ideal output voltages. And the errors Δ*V_out_*_1_ were 6.1%, 4.3% and 2.7%, respectively. The offset correction current source has a positive improvement on the output deviation generated by the offset. And the correction ratios can reach 60.9%, 69.8% and 79.1%.

Because the value of the current *I* is regulated by only three groups of transistors as shown in Figure 4, so the outputs are roughly equal to the ideal values. If there are more groups of transistors and compensation current sources, the output will be more close to the ideal value. But taking into account that the correction circuit with three groups of compensation current sources has been able to meet the design requirements, we do not introduce more compensation current sources.

## 4. Tracking Circuit

**Figure 6.**
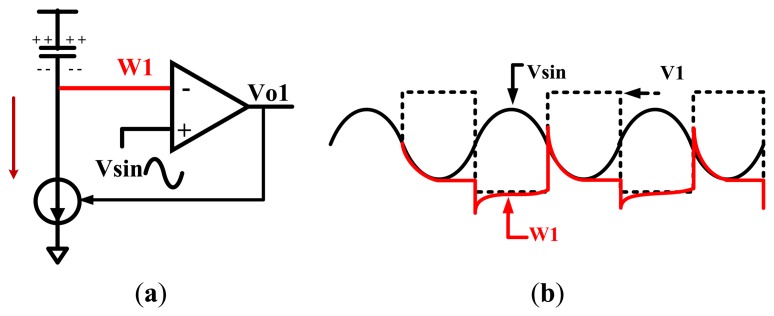
(**a**) Tracking circuit in the trough tracking state. (**b**) When *V*_1_ is high, tracking circuit tries to detect the input trough.

**Figure 7.**
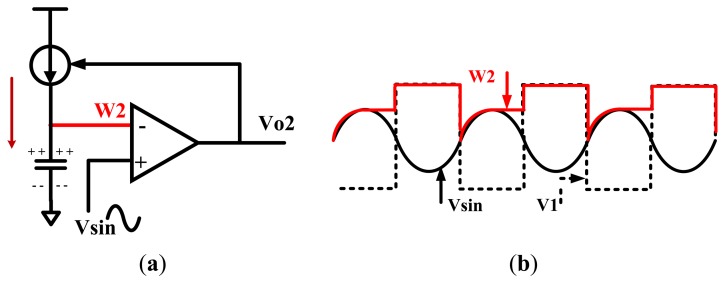
(**a**) Tracking circuit in the crest tracking state. (**b**) When *V*_1_ is low, the tracking circuit tries to detect the input crest.

The ideal wave of the F2F encoding is rectangular wave. However, the input of the magnetic sensor chip is actually similar to sine wave, or triangle wave. Consequently, the AGC output is also similar to sine wave, or triangle wave. Therefore, the key to F2F decoding is the accurate localization of crests and troughs of the AGC output. When the waveform rises from the trough to the crest, the slope of the waveform is positive. As the input level drops from the crest to the trough, the slope is negative. The slope changes from negative to positive in the trough. The proposed tracking circuit can detect the changing of the slope and find out the crests and troughs. This tracking circuit generates a trough found signal when the slope changes from negative to positive, and turns into the crest tracking state when the slope has been positive and the input is above *V_sta_* + 100 mV. On the other hand, it generates a crest found signal when the slope changes from positive to negative and turns into the trough tracking state when the slope has been negative and the input is under *V_sta_* - 100 mV. In other words, track circuit has two operation states, and can switch between these two states based on the slope changing and the input level. A digital signal *V*_1_ is used to control this selection. When *V*_1_ is high, the tracking circuit turns into the trough tracking state. The circuit begins to track the trough of the sine wave *V*_sin_ (shown as Figure 6). When *V*_1_ is low, the tracking circuit turns into the crest tracking state to track the crest of the sine wave *V*_sin_ (shown as Figure 7).

### 4.1. Trough Tracking State

When *V*_1_ is high, as shown in Figure 6, line *W*_1_ begins to track the wave *V*_sin_ slowly. When *V*_sin_ changes from low to high, *W*_1_ becomes lower than *V*_sin_ because the output voltage *V_o_*_1_ becomes high and the current source will be cut off. *V*_1_ becomes low when the input *V*_sin_ is above *V_sta_* + 100 mV, forcing the tracking circuit turns into the crest tracking state. This additional 100 mV is used to remove the false crest, so as to improve the noise performance. If there is no noise in the input, then the additional 100 mV is unnecessary. The waveform in this ideal case is depicted in Figure 6b.

### 4.2. Crest Tracking State

When *V*_1_ is low, the tracking circuit is in the crest tracking state, as shown in Figure 7. Line *W*_2_ begins to track the input *V*_sin_ slowly. When *V*_sin_ changes from high to low, output voltage *V_o_*_2_ becomes low and the current source will be cut off. Therefore, the level of *W*_2_ remains unchanged. *V*_1_ becomes high when the input *V*_sin_ is under *V_sta_* - 100 mV, letting the tracking circuit turns into the trough tracking state. The additional 100 mV can avoid the decoding error caused by small spikes. If there are no small spikes in the input, the additional 100 mV is also unnecessary. The waveform in this ideal case is depicted in Figure 7b.

### 4.3. Difference between the Trough Tracking and Crest Tracking States

**Figure 8.**
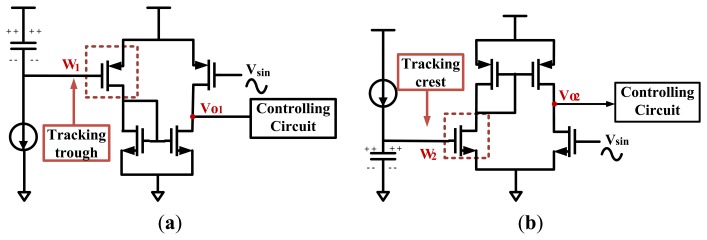
(**a**) Details of the tracking circuit in the trough tracking state. (**b**) Details of the tracking circuit in the crest tracking state.

Figure 8 describes the details of the tracking circuit in the trough tracking and crest tracking states, respectively. Line *W*_1_ is an input of a PMOS transistor (as shown in the red dashed box of Figure 8a). Since PMOS is in the saturation region when the input *W*_1_ is low, this structure can track the trough even the input is quite low. Similarly, line *W*_2_ is an input of an NMOS transistor in the crest tracking state (as shown in the red dashed box of Figure 8b). The NMOS is in the saturation region when the input *W*_2_ is high enough, thus it can increase the ability of tracking crest. All in all, choosing different amplifier structures in the trough tracking and crest tracking states can increase the signal sensing range.

### 4.4. Simulation of the Tracking Circuit

In order to verify the principle of operation, the tracking circuit is simulated in Cadence Spectre. The setting of the simulation is as following Table 3.

**Figure 9.**
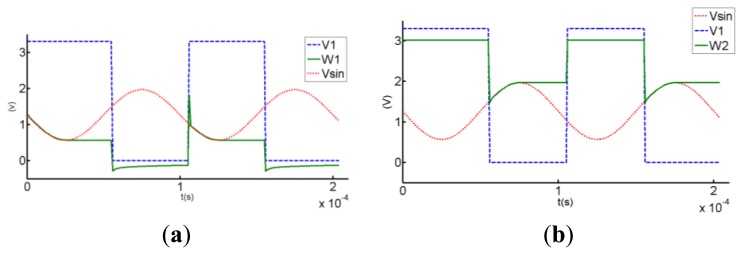
(**a**) Simulation waveform of tracking circuit in the trough tracking state. (**b**) Simulation waveform of tracking circuit in the crest tracking state.

*V*_1_ is provided as a rectangular wave, while *V*_sin_ is a sine wave input. The simulation waveforms of the tracking circuit are shown as Figure 9. Comparing the simulation waveforms shown in Figure 9a,b with the ideal waveform curve shown as Figures 6b and 7b, respectively, the shapes are almost the same, except that *V*_1_ becomes high when the input *V*_sin_ is lower than *V_sta_* - 100 mV and *V*_1_ becomes low when the input *V*_sin_ is higher than *V_sta_* + 100 mV. The additional 100 mV is introduced to decrease the decoding error caused by small spikes. Figure 9a shows the simulation curves of the trough tracking state. When *V*_1_ is high, *W*_1_ begins to track the trough; When *V*_1_ turns low, circuit starts to track the crest. Figure 9b displays the simulation curves of the crest tracking state.

## 5. Measurement

**Figure 10.**
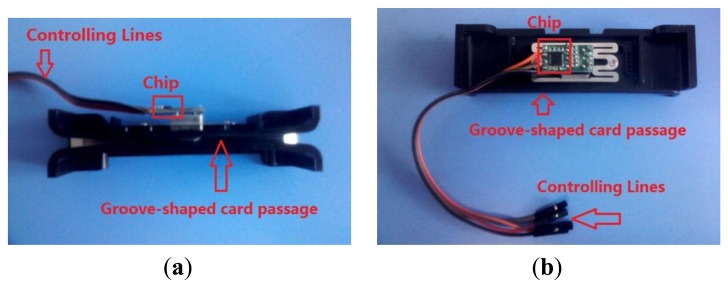
(**a**) Top view of the line-controlling magnetic card swiping system. (**b**) Side view of the line-controlling magnetic card swiping system.

**Figure 11.**
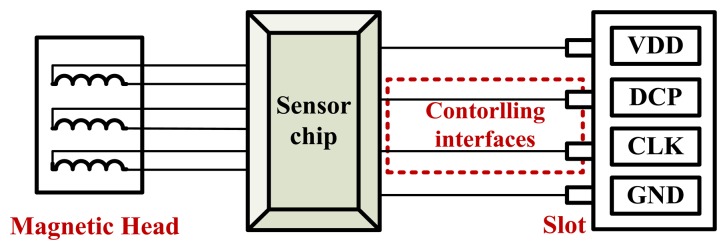
The block diagram of the card swipe machine.

Figure 10a,b are the actual photos of the swiping system presented from top and side. Figure 11 is the block diagram of the line-controlling magnetic card swiping system.

As the figures shown, one side of the chip is connected to the magnetic heads, and the other side is connected to a four-hole slot. The four-hole slot can be used to connect to a micro-processor.

**Figure 12.**
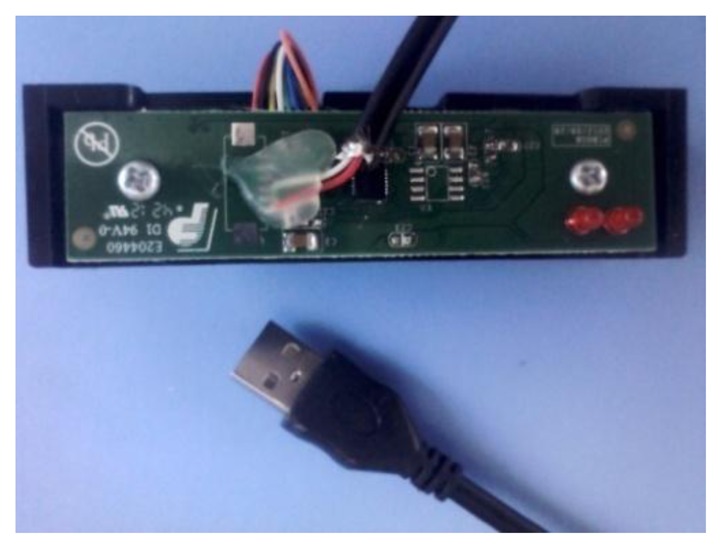
Photo of magnetic card swiping system with USB's interface.

When the magnetic card is swiped at a certain speed (10–200 cm/s) along a groove-shaped card passage, the magnetic stripe on the card will contact with the read heads in the card passage. The signals are read from a magnetic data carrier, e.g., a card carrying magnetic stripe, with the help of a magnetic read head. The two-frequency data signal, also known as a bi-phase or F2F signal, is accurately decoded by amplifying and digitizing. The micro-processor is connected with the chip through the controlling interfaces DCP and CLK. In addition to the line-controlling magnetic card swiping system mentioned above, we also designed a magnetic card swiping system with a USB interface (as shown in Figure 12).

**Figure 13.**
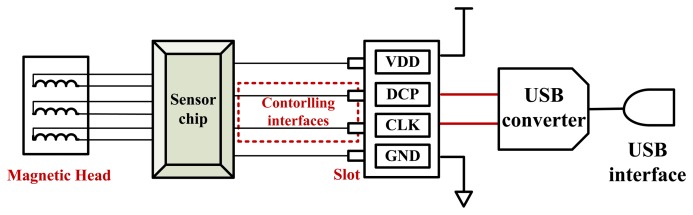
The block diagram of magnetic card swipe machine with USB's interface.

The principle is basically the same as the line-controlling ones. The only difference is that an USB converter is added between the chip and the USB interface, as depicted in Figure 13. The additional USB converter makes it easier to test the performance of the sensor chip. It can obtain the card information from the terminal through the USB interface directly.

**Figure 14.**
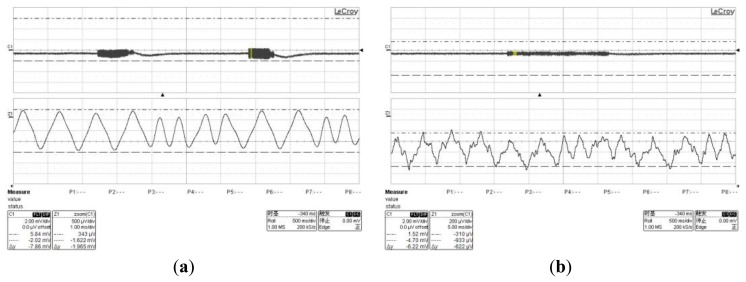
(**a**) The sensed signals of fast swiping. (**b**) The sensed signals of slow swiping.

We test the performance of the sensor chip. The chip is able to work properly and fully comply with the design requirements. It consumes less than 120 μA, when the sensor chip is held in the sleep state. The sensed signals of fast swiping and slow swiping are shown in the following figure (Figure 14).

According to the F2F encoding, the sensed signals can be read out as a sequence of binary signals “00000001101000001”. When the swipe speed was slow, there were some small spikes in the sensed signals, as Figure 14b shown. However, due to the proposed tracking circuit and AGC with offset correction circuit, the binary signal still could be read out.

## 6. Conclusions

The proposed high performance and low power consumption magnetic card sensor chip implemented in 0.35 μm technology can simultaneously detect, decode and read out three tracks information in a magnetic data carrier. It consists of a reference voltage generating circuit, a filter circuit, a power-on reset circuit and a pre-decoding circuit module, *etc.* Based on the foundation of traditional decode and sensor chip, offset correction circuit and tracking circuit are proposed to improve the decoding accuracy and expand the sensing range of the signals. The circuit modules were implemented in 0.35 μm TSMC technology, and measurement results showed that the sensor chip fully met the design specifications.

## Figures and Tables

**Table 1.** The differences of these five chips.

**Chip Name**	**Num of Pins**	**Num of R**	**Num of C**	**Num of Tracks**
BS730	24	0	4	3
AIS-2103N	32	6	8	3
SMR200	24	6	8	2
M3-2200G	28	13	11	2
Chip Proposed in This paper	8	0	0	3

**Table 2.** Performance of the correction circuit.

**Results**	*V_out_*_1_**(without Offset Correction)**	Δ*V_out_*_1_	*V_out_*_2_**(with Offset Correction)**	Δ*V_out_*_2_	**Correction Ratio** $\frac{\Delta V_{\text{out}1}-\Delta V_{\text{out}2}}{\Delta V_{\text{out}1}}$

*V_in_*					
1.15 V	1.329 V	15.5%	1.080 V	6.1%	60.9%
1.27 V	1.449 V	14.1%	1.216 V	4.3%	69.8%
1.39 V	1.569 V	12.9%	1.352 V	2.7%	79.1%

**Table 3.** Simulation setting.

*V*_sin_ (sine wave)	Offset Voltage	1.27 V	Amplitude	0.7 V	Frequency	10 kHz

*V*_1_ (square wave)	Voltage1	3.3 V	Voltage2	0 V	Pulse width	50 μs
